# *In vivo* antiplasmodial and toxicological effect of crude ethanol extract of *Echinops kebericho* traditionally used in treatment of malaria in Ethiopia

**DOI:** 10.1186/s12936-015-0716-1

**Published:** 2015-05-10

**Authors:** Alemayehu Toma, Serawit Deyno, Abrham Fikru, Amalework Eyado, Andrew Beale

**Affiliations:** Pharmacology Unit, School of Medicine, Hawassa University, PO Box 1560, Hawassa, Ethiopia; Biomedical team, School of Veterinary Medicine, Hawassa University, Hawassa, Ethiopia; Department of Biology, College of Natural and Computational Sciences, Addis Ababa University, Addis Ababa, Ethiopia; Independent scholar, AuthorAID Mentor, Milange, Mozambique

**Keywords:** Anti-malarial activity, *Echinops kebericho*, Parasitaemia, *Plasmodium berghei*

## Abstract

**Background:**

Medicinal plants have contributed significantly to current malaria treatment. Emergence of resistance to currently available drugs has necessitated the search for new plant-based anti-malarial agents and several plant-based, pharmacologically active anti-malarial compounds have been isolated. This study was conducted to validate the traditional usage of *Echinops kebericho* for treating malaria in the traditional health care system of Ethiopia.

**Methods:**

The roots of *E. kebericho* were collected from Masha Woreda, Sheka Zone. After collection, the plant materials were identified by a taxonomist, dried under shade and crushed to powder for extraction. The powdered roots were extracted by maceration using 70 % ethanol. Acute toxicity study of the crude extract was carried out in Swiss albino mice. The *in vivo* anti-malarial activity of plant extract (200, 350 and 500 mg/kg) of *E. kebericho* roots against a chloroquine (CQ) sensitive strain of *Plasmodium berghei* strain ANKA was assessed using the four-day suppressive test procedure. Parameters such as parasitaemia, packed cell volume, body weight and survival time were then determined using standard tests.

**Results:**

Oral administration of the ethanol extract showed significant (P <0.001) parasitaemia suppression at dose levels of 350 and 500 mg/kg in dose-related manner compared with the negative control. Five hundred mg/kg showed the highest (57.29 ± 1.76 %) parasitaemia suppression. The survival times of *P. berghei*-infected mice were also increased in a dose-dependent manner but the test material did not prevent weight loss associated with increased parasitaemia. The result also showed the plant material prevented the loss in packed cell volume associated with increased parasitaemia. Its oral LD_50_ was found to be greater than 5,000 mg/kg, indicating its wider safety margin in mice.

**Conclusion:**

The result revealed the ethanol extract of *E. kebericho* roots has anti-malarial activity against *P. berghei* in an animal model and lends support to the use of the plant to combat malaria in Ethiopian folk medicine. Further work is necessary to isolate, identify and characterize the active principles from the plant material.

## Background

Malaria, one of the oldest human diseases, has become the main concern of the World Health Organization (WHO) in the past few decades, not only as a result of its re-emergence as the biggest infectious killer, but also expansion of its distribution to previously non-affected areas [1]. In Ethiopia, for example, the lowlands have always been regarded as areas of high malaria transmission, however, this appears to be changing due to climatic and ecological changes [2, 3]. Recently the epidemic spread into the highland areas where a large proportion of the population lives [4].

The resurgence of the disease due to drug-resistant strains of the parasite and insecticide-resistant strains of the mosquito vector catches the attention of many scholars. As a result of which, there have been various efforts to combat the problem of parasite resistance, including reversing chloroquine resistance, use of combination therapy, and discovery of new anti-malarial compounds from various sources, especially from traditional medicinal plants [5, 6].

Traditional medicinal plants have contributed significantly to current malaria treatment. The first effective drug treatment against malaria was quinine, which was extracted from the Cinchona tree. The chemical structure of quinine was used to synthesize new anti-malarial drugs, such as chloroquine and primaquine. The importance of plants as effective anti-malarial was further reinforced by the isolation of artemisinin from the Chinese medicinal plant, *Artemisia annua *(Family *Asteraceae*). Artemisinins are presently the most effective drug against multi-drug resistant strains of *Plasmodium falciparum*. Several pharmacologically active anti-malarial compounds have been in development from East African medicinal plants including Ethiopia [7].

The genus *Echinops* is reported to comprise over 120 species, of which four (*Echinops kebericho* Mesfin*, Echinops buhaitensis, Echinops ellenbeckii, and Echinops longisetus*) are confined to the Ethiopian highlands. *Echinops kebericho *(local Amharic name: *kebercho*) is traditionally used for the treatment of fever, diarrhoea, malaria, as taenicide, stomach ache, and typhus [8–11]. Moreover, the smoke from burning the plant is inhaled to relieve headache and as a cure for “evil eye” (possession by evil spirits in Ethiopian folk religion). The smoke is inhaled to fight typhus and fever, and is known to be used as a fumigant for mosquitoes and as a snake repellent. The traditionally used preparation varies among society. Inhalation for pain relief and oral chewed preparation for malaria, diarrhoea and stomach ache among the different preparation used in traditional medicine [9, 10]. Extracts and essential oils of the roots of *E. kebericho* have been assessed for their antimicrobial, antihelmintic and molluscicidal activities [12]. Although the plant is in use for the treatment of malaria in Ethiopian society, there is no laboratory-based evidence for the effectiveness and safety of the plant. This study is, therefore, designed to evaluate the *in vivo* anti-malarial activity and acute toxicity test of the ethanol extract of *E. kebericho.*

## Methods

### Chemicals

The chemicals used were absolute ethanol (Reagent Chemical-Ltd, China), Giemsa stain 10 % (Shenyang Xin Guang, China), chloroquine phosphate (Ethiopian Pharmaceutical Manufacturing, Ethiopia).

### Collections and preparation of plant materials

The roots of *E. kebericho* were collected from Masha Woreda, Sheka Zone South Nation’s Nationalities and Peoples Region. After collection, the plant materials were identified by a taxonomist and a voucher specimen (AL 001) was deposited at Addis Ababa University (AAU) Herbarium, dried under shade and crushed to powder for extraction.

### Extraction

The powdered roots were extracted by maceration using 70 % ethanol. Next, the mixture was filtered using Whatmann filter paper No. 1. The filtered extract was made ethanol-free by evaporating it using rotary evaporator under reduced pressure. The actual yield expressed in weight by weight (w/w) was 13.2 %. The filtrate obtained was kept refrigerated at 8 °C and fresh solution was prepared for each experiment by using 3 % tween 80.

### Laboratory animal preparation

Healthy adult male Swiss albino mice weighing 25–32 g at six to eight weeks age were used in this study. All animals were housed in standard cages in the animal house and fed a standard pellet diet and tap water *ad libitum*. The test animals were acclimatized for two weeks and put randomly in to five groups.

### The parasite strain

The anti-malarial activity of the extract of *E. kebericho* was tested using infected mice with chloroquine-sensitive *Plasmodium berghei* strain ANKA, which are maintained at the Animal House of the Department Biomedical Sciences, AAU. The parasites were maintained by serial passage from infected mice to non-infected mice on weekly basis to maintain viability of the strain.

#### *In vivo* anti-malarial test

Antiplasmodial activity of the test extract was performed in a four-day suppressive standard test [13]. An infected blood sample was collected from the heart of a donor mouse with a rising parasitaemia of about 30 %. The blood was diluted with normal saline with the intention that each 0.2 ml contained approximately 1 × 10^7^*P. berghei*-parasitized erythrocytes. Male Swiss albino mice weighing 25–32 g. were inoculated on first day (D0), intraperitoneally, with 0.2 ml of infected blood. The mice were then divided randomly into five groups of five mice each for each group. Three groups of animals were assigned as the test groups and the other two groups were used as control (positive and negative). Three hours after infection, the three test groups were orally administered, with 200, 350 and 500 mg/kg/day doses of the root extract. Chloroquine at the dose of 25 mg/kg/day (oral) and an equivalent volume of vehicle (3 % of Tween 80) was administered to the positive and negative control groups, respectively, for four consecutive days (D0 to D3). This was done for each group. On the fifth day (D4), thin smears were made from the tail blood of each mouse, fixed with methanol and stained with 10 % Giemsa. The parasitaemia level was determined by counting the number of parasitized erythrocytes out of 100 erythrocytes in random fields of the microscope. Average percentage parasitaemia was calculated using the formula:

$$ \%\ \mathrm{Parasitaemia}=\kern1em \frac{\mathrm{Number}\;\mathrm{of}\;\mathrm{infected}\;\mathrm{RBCs}}{\mathrm{Total}\;\mathrm{number}\;\mathrm{of}\;\mathrm{RBCs}}\kern0.5em \times \kern0.5em 100 $$

Average percentage of chemosuppression was calculated using the formula:

$$ \%\;\mathrm{Suppression} = \kern1em \frac{\mathrm{Parasitaemia}\ \mathrm{in}\ \mathrm{negative}\ \mathrm{control} - \mathrm{Parasitaemia}\;\mathrm{in}\;\mathrm{test}\;\mathrm{group}}{\mathrm{Parasitaemia}\;\mathrm{in}\;\mathrm{negative}\;\mathrm{control}}\kern0.5em \times \kern0.5em 100 $$

The body weights and packed cell volume (PCV) of the mice were taken to observe whether the test extract prevented the weight loss and reduction in PCV that are commonly observed with increasing parasitaemia in infected mice. The weight and PCV were taken at D0 and D4. The PCV was determined by the following equation

$$ \mathrm{P}\mathrm{C}\mathrm{V}=\kern1em \frac{\mathrm{Volume}\kern0.5em \mathrm{of}\kern0.5em \mathrm{the}\kern0.5em \mathrm{total}\kern0.5em \mathrm{erythrocytes}\kern0.5em \mathrm{in}\kern0.5em \mathrm{a}\kern0.5em \mathrm{given}\kern0.5em \mathrm{volume}\kern0.5em \mathrm{of}\kern0.5em \mathrm{blood}}{\mathrm{Total}\kern0.5em \mathrm{blood}\kern0.5em \mathrm{volume}}\kern0.5em \times \kern0.5em 100 $$

### Determination of the mean survival time

Mortality was monitored daily and the number of the days from the time of inoculation of the parasite up to death was recorded for each mouse in the treatment and control groups throughout the follow-up period. The mean survival time (MST) for each mouse was recorded after the treatment periods.

### Test for acute oral toxicity studies

Acute toxicity study was performed on mice of either sex selected at random. The animals were fasted overnight and provided with only water. They were then divided into four groups, six animals in each group (three male and three female), after which the fraction was administered orally at increasing dose level of 2,000, 3,500, 5,000 mg/kg via oral gavage as per guidelines suggested by the Organization for Economic Cooperation and Development (OECD) [14]. Animals were kept under close observation for four hours after administering the extract for behavioural, neurological and autonomic profile and observed for any change in general behaviour and/or other physical activities and mortality within 24 hours.

### Statistical analysis

The results were presented as the mean ± SEM (standard error of mean) for each group of experiments. Data on parasitaemia, body weight, PCV, and survival times were analysed using Windows SPSS Version 20. Statistical significance was determined by one-way analysis of variance (ANOVA), and independent t-tests. All data were analysed at a 95 % confidence interval.

## Results

### Acute oral toxicity test

Acute toxicity studies conducted revealed that the administration of graded doses of hydro-alcoholic extract of *E. kebericho* (up to a dose of 5,000 mg/kg) did not produce significant changes in behaviours, such as alertness, motor activity, breathing, restlessness, diarrhoea, convulsions, coma, and appearance of the animals. No death was observed up to the dose of 5 g/kg body weight, indicating that the medium lethal dose (LD_50_) could be greater than 5 g/kg body weight in mice. The mice were physically active. These effects were observed during the experimental period.

The *in vivo* antiplasmodial activity study revealed that the ethanol extract of the roots of *E. kebericho* produced the highest chemosuppression in a dose-dependent manner as compared to the negative control in this study. The chemosuppression was 16.93 ± 1.17 %, 29.46 ± 1.93 % and 57.29 ± 1.76 % for 200, 350, 500 mg/kg/day doses, respectively (Table 1). The chemosuppressive effect produced by doses beyond 350 mg/kg was very significant (P <0.001) compared with the negative control.

**Table 1:** Anti-malarial activity of different doses of *Echinops kebericho* roots against *Plasmodium berghei* in Swiss albino mice

Treatment groups	% survival of animal on day 10	% parasitaemia	% chemosuppression
Negative control	72.30 ± 2.37	62.40 ± 2.94	00.00 ± 0.00
200 mg/kg EKM	77.20 ± 2.51	51.83 ± 4.93	16.93 ± 1.17
350 mg/kg EKM	81.50 ± 1.40	44.02 ± 0.72*	29.46 ± 1.93
500 mg/kg EKM	95.80 ± 1.86*	26.65 ± 1.01*	57.29 ± 1.76
25 mg/kg CQ	100.00 ± 0.00*	00.00 ± 0.00*	100.00 ± 0.00

Results are expressed as means ± S E M, n = 5. *P < 0.001 versus negative control

Comparison of the mean survival time of mice in the experimental groups with the untreated group for all test groups was performed (Table 1). The result indicated that mice treated with all doses of extract lived longer than the negative control. All concentrations of extract employed in this study have no significant prevention effect on weight loss of mice at all dose levels (p >0.05) compared with the control group (Table 2). All the test extracts employed in this study have significantly prevented PCV loss of mice at all dose levels (P <0.001) compared with the control group (Table 3).

**Table 2:** Body weight of *Plasmodium berghei*-infected mice before and after the administration of the test extract of roots of *Echinops kebericho*

Treatment groups	Before treatment	Weight (g) after treatment	% change
Negative control	29.14 ± 0.26	28.64 ± 0.21	−1.72 ± 0.24
200 mg/kg EKM	28.90 ± 0.18	27.62 ± 0.40	−4.43 ± 0.22
350 mg/kg EKM	29.28 ± 0.27	28.12 ± 0.31	−3.96 ± 0.28
500 mg/kg EKM	29.06 ± 0.18	28.46 ± 0.30	−2.06 ± 0.21
25 mg/kg CQ	28.74 ± 0.11	31.08 ± 0.27*	+8.14 ± 0.16

Results are expressed as means ± S E M, n = 5. *P <0.001 versus negative control

**Table 3:** Packed cell volume (PCV) of *Plasmodium berghei*-infected mice before and after the administration of the test extract of roots of *Echinops kebericho*

Treatment groups	Before treatment	PCV (%) after treatment	% change
Negative control	50.34 ± 0.93	40.68 ± 0.91	−19.12 ± 0.14
200 mg/kg EKM	52.78 ± 1.58	50.34 ± 1.51*	−4.62 ± 1.11
350 mg/kg EKM	53.26 ± 1.52	52.34 ± 1.50*	−1.73 ± 1.15
500 mg/kg EKM	51.73 ± 0.48	49.41 ± 0.64*	−4.48 ± 0.52
25 mg/kg CQ	50.40 ± 0.39	52.30 ± 0.45*	+3.77 ± 0.42

Results are expressed as means ± S E M, n = 5. *P < 0.001 versus negative control

## Discussion

Plant products are frequently considered to be less toxic and have fewer adverse effects than synthetic ones. A growing number of peoples are therefore turning to alternative therapy, including plant medicines. Traditional Chinese medicine has been used in clinical practice for several centuries. However, the compounds and precise mechanisms of most plant medicines remain to be determined.

*Plasmodium* species that cause human disease are essentially unable to infect non primate animal models. Therefore, for the *in vivo* evaluation of anti-malarial compounds in rodents, the rodent malaria parasite is employed, due to the sensitivity of *P. berghei* parasite to chloroquine [15]. A four-day suppressive test was used to assess the efficacy of the extract by comparison of blood parasitaemia and mouse survival time in treated and untreated mice.

The evaluation of anti-plasmodial activity of hydro-ethanolic root extract of *E. kebericho* on early malaria infection, in a four-day suppressive test, shows a statistically significant parasitaemia suppression of 57.29 ± 1.76 % at the highest dose, with the longest survival time compared to all other doses and the negative control. By showing the highest parasitaemia, chemosuppression and longest survival time, it suggests that 500 mg/kg body weight of extract might be the optimal therapeutic dose in mice. The prolonged MST of the mice indicates that the extract suppressed *P. berghei* and reduced the overall pathologic effect of the parasite. The decrease in parasitaemia with increasing concentration of the extract reflects an inhibitory activity on parasite replication. These results indicate significant anti-malarial potential for isolating a purer compound [16–18].

In a four-day suppressive test, it was only chloroquine-treated mice that prevented body weight loss significantly (P <0.001). No extract-treated mice showed significant (P >0.05) prevention against body weight loss when compared to negative control. Body weight loss in extract-treated mice might possibly be due to a depressing effect of the crude extract on food intake or appetite. This result is in agreement with that of a previous study on other medicinal plants [17, 18].

*Plasmodium* infection is correlated with the incidence of high destruction of red blood cells. The anaemia that may result can be life threatening [18, 19]. During the four-day suppressive test, the effect of the hydro-ethanolic root extract of *E. kebericho* on the PCV was tested to assess the reversing effect of the extract from infection induced anaemia. The 200, 350 and 500 mg/kg body weight of extract showed significant prevention against PCV reduction (p <0.001). This may be due to the effect of the extract in preventing PCV on early infection. Chloroquine showed a strong protective effect in preventing PCV reduction compared to extract-treated groups and negative control.

Another observation drawn from this study is the relative safety of the hydro-ethanolic extract of *E. kebericho* roots at the graded dose of up to 5,000 mg/kg. According to Garner and coworkers, any compound or drug with an oral LD_50_ estimate greater than 1,000 mg/kg could be considered low toxic and safe [20]. Arising from this, the *E. kebericho* at an oral dose of 5,000 mg/kg can be considered relatively safe on acute exposure.

## Conclusion

This study demonstrated the anti-malarial activity of ethanol extract of *E. kebericho* roots against *P. berghei* in an animal model. The findings of this study confirm the traditional usage of the plant to combat malaria in Ethiopian folk medicine. These results suggest that it is possible to isolate active anti-malarial compounds from the extract.
